# Bioinformatically predicted deleterious mutations reveal complementation in the interior spruce hybrid complex

**DOI:** 10.1186/s12864-017-4344-8

**Published:** 2017-12-15

**Authors:** Gina L. Conte, Kathryn A. Hodgins, Sam Yeaman, Jon C. Degner, Sally N. Aitken, Loren H. Rieseberg, Michael C. Whitlock

**Affiliations:** 1 Department of Forest and Conservation Sciences, University of British Columbia, 3041-2424 Main Mall, Vancouver, BC V6T 1Z4 Canada; 2 Department of Botany, University of British Columbia, 3200-6270 University Blvd, Vancouver, BC V6T 1Z4 Canada; 3 Department of Zoology, University of British Columbia, 4200-6270 University Blvd, Vancouver, BC V6T 1Z4 Canada; 4 Present Address: School of Biological Sciences, Monash University, Clayton Campus, Melbourne, Victoria 3800 Australia; 5 Present Address: Department of Biological Sciences, University of Calgary, 2500 University Dr NW, Calgary, AB T2N 1N4 Canada

**Keywords:** Deleterious mutations, Mutation load, Complementation, Hybridization, Population genomics, Conifers, Spruce

## Abstract

**Background:**

Mutation load is expected to be reduced in hybrids via complementation of deleterious alleles. While local adaptation of hybrids confounds phenotypic tests for reduced mutation load, it may be possible to assess variation in load by analyzing the distribution of putatively deleterious alleles. Here, we use this approach in the interior spruce (*Picea glauca* x *P. engelmannii*) hybrid complex, a group likely to suffer from high mutation load and in which hybrids exhibit local adaptation to intermediate conditions. We used PROVEAN to bioinformatically predict whether non-synonymous alleles are deleterious, based on conservation of the position and abnormality of the amino acid change.

**Results:**

As expected, we found that predicted deleterious alleles were at lower average allele frequencies than alleles not predicted to be deleterious. We were unable to detect a phenotypic effect on juvenile growth rate of the many rare alleles predicted to be deleterious. Both the proportion of alleles predicted to be deleterious and the proportion of loci homozygous for predicted deleterious alleles were higher in *P. engelmannii* (Engelmann spruce) than in *P. glauca* (white spruce), due to higher diversity and frequencies of rare alleles in Engelmann. Relative to parental species, the proportion of alleles predicted to be deleterious was intermediate in hybrids, and the proportion of loci homozygous for predicted deleterious alleles was lowest.

**Conclusion:**

Given that most deleterious alleles are recessive, this suggests that mutation load is reduced in hybrids due to complementation of deleterious alleles. This effect may enhance the fitness of hybrids.

**Electronic supplementary material:**

The online version of this article (10.1186/s12864-017-4344-8) contains supplementary material, which is available to authorized users.

## Background

Mutation load is the reduction in fitness caused by deleterious alleles segregating at mutation-selection balance in populations [1, 2]. Decreased mutation load in hybrids may increase their fitness relative to parents [3–5]. Because mutations are random and deleterious alleles may rise in frequency through genetic drift, mutation load in partially reproductively isolated groups is likely to result from largely distinct sets of alleles [6]. Therefore, relative to parents, mutation load due to additive deleterious alleles should be intermediate in hybrids due to their intermediate number of deleterious alleles. However, most deleterious alleles are thought to be at least partially recessive [2, 7–9], and mutation load due to recessive deleterious alleles should be lower in hybrids than in parental species due to their lower homozygosity of deleterious alleles. This latter effect is known as complementation and is the mechanism underlying the dominance hypothesis of heterosis [3, 4].

Reduction of mutation load in hybrids may commonly contribute to hybrid zone dynamics. However, the possibility that hybrids are also locally adapted to environmental conditions in contact zones confounds our ability to phenotypically detect reduced mutation load. In the case of bounded hybrid superiority, hybrids are predicted to be more fit than parents in their own environment and less fit in parental environments, a clear signal of local adaptation [10]. However, since we have no theoretical expectation for the precise differences in fitness between hybrids and parents in each environment that are caused by local adaptation, it is difficult to tell whether reduced mutation load has an additional effect on hybrid fitness, which should enhance their fitness across all environments.

Here we introduce an alternative approach to assess whether mutation load is reduced in hybrid zones, which identifies bioinformatically predicted deleterious alleles that are segregating in populations and then compares properties of these alleles in hybrid and non-hybrid individuals. Traditionally, identifying the alleles underlying mutation load in natural populations has been very difficult. Most deleterious alleles contributing to mutation load tend to be of small effect and are kept at low frequency by purifying selection [2, 11], making their individual effects on fitness or phenotype too small to detect with reasonable sample sizes. With the diversity of genomic data available today, it is now possible to use protein conservation to predict whether nonsynonymous alleles are likely to be deleterious. Generally, alleles are thought to be more deleterious when they involve either a nonsynonymous change in a phylogenetically conserved position or a change to a substantially different amino acid (inferred based on substitution probabilities or biochemical qualities). Several methods have been developed that implement this approach, including GERP [12], MAPP [13], SIFT [14], PolyPhen-2 [15] and PROVEAN [16]. While not all alleles predicted to be deleterious by these methods will actually be so, and while some deleterious alleles may be missed, tests of functionally verified deleterious alleles have typically found both specificity and sensitivity to be between 70 and 90% [15–17] though specificity was lower in [18]) (see the Discussion regarding accuracy of specificity estimates). Furthermore, a handful of studies have now applied these methods to genomic datasets, finding that alleles predicted to be deleterious are at lower average allele frequencies than those predicted to not be deleterious, consistent with the expected effect of purifying selection [17, 19–25]. By providing insight into the genetic basis of mutation load, these methods offer a means for studying load in a comparative manner. Thus far, few studies employing these methods at a genome-wide scale have focused on natural populations and fewer still have investigated natural hybrid zones.

The interior spruce hybrid complex is an ideal natural system to investigate the patterns of mutation load in hybridizing species. The complex is composed of *Picea glauca* (Moench) Voss (white spruce), *Picea engelmannii* Parry ex Engelm. (Engelmann spruce), and their hybrids. White spruce is a boreal species with a widespread continuous range across Canada and Alaska [26]. Rapid postglacial northwestern spread in the western interior of Canada has led to low chloroplast DNA diversity in white spruce populations west of the great lakes and east of Alaska [26, 27]. Engelmann spruce is a sub-alpine species with a fragmented range in western North America [28]. Populations in western Canada are the result of northward post-glacial expansion [29]. Contrary to expectations, isozyme diversity of Engelmann spruce increases with latitude, apparently due to hybridization with white spruce [29]. The two species hybridize extensively where their ranges overlap in western Canada. Hybrids occupy intermediate ecological and elevational niches [30], and they appear to be fitter than parental species in these intermediate habitats, supporting the bounded superiority model of hybrid zone maintenance [31]. We predict that these hybrids should also benefit from decreased mutation load. However, the observed local adaptation of hybrids to intermediate environments hampers our ability to determine this at the phenotypic level. Instead, we can test for a signature of reduced mutation load in hybrids at the genetic level.

Conifers, in general, are known to suffer from high mutation load [32–34], and a reduction in load via hybridization may provide a substantial fitness benefit. Previous estimates of mutation load in conifers have been derived by comparing viable seed numbers per cone from self-pollination with those from unrelated crossings. The number of lethal equivalents per zygote is estimated using 2*B* = −4ln*R*, where *B* is the average number of lethal equivalents per gamete and *R* is the ratio of percent selfing survivors to percent outcross survivors [7, 34]. The estimated number of lethal equivalents in white spruce, 12.6 per diploid individual (affecting seed yield, germination, and survival to age 17 years), is one of the highest estimated in conifers or in any life form [35]. However, the deleterious alleles underlying their high mutation load have yet to be identified. Furthermore, mutation load has not been estimated in Engelmann spruce or in white-Engelmann hybrids. By identifying deleterious alleles in white spruce, Engelmann spruce and their hybrids, we are able to infer the relative severity of mutation load in the two species and their hybrids, and to better understand the effects of hybridization on load.

We used the program PROVEAN [16] to identify putative deleterious alleles within a previously reported exome capture dataset [36]. We then determined the proportion of alleles that are putatively deleterious in individuals of each of the two spruce species and in hybrids, as well as the proportions of loci that are heterozygous or homozygous for putative deleterious alleles. This information allowed us to test the following hypotheses: first, if putative deleterious alleles are actually deleterious, then we predict that they should occur at lower frequencies than non-deleterious alleles and be associated with a decrease in fitness (via a phenotypic fitness proxy); and second, we hypothesize that mutation load should be reduced in hybrids.

## Methods

### Data collection

We used a previously reported exome capture dataset containing about nine million single nucleotide polymorphisms (SNPs) identified in 579 spruce individuals from 254 locations in British Columbia and Alberta, Canada, and grown in a common garden experiment [36, 37]. Methods for sample collection and growth, and for SNP identification are documented therein. Briefly, for all sequence alignment and downstream analysis, we used the February 2013 version of the white spruce genome (SMarTForests Project [38]). Sequenced reads were filtered and trimmed using the FASTX toolkit (http://hannonlab.cshl.edu/fastx_toolkit/index.html). We aligned the remaining reads to the draft genome using the Burrows-Wheeler Aligner mem algorithm [39] marked and removed polymerase chain reaction (PCR) duplicates using Picard MarkDuplicates (http://broadinstitute.github.io/picard/), and performed realignment around indels using GATK’s IndelRealigner [40]. Base Quality Score Recalibration (GATK) was conducted using intermediate SNP databases. Following recalibration, a working set of SNPs was called using GATK-Unified Genotyper (v3.3.0 [40]), with any genotypes that did not have individual quality scores >20 and depth > 5× set to ‘N’ (i.e. missing data). The set of SNPs were filtered to eliminate any loci that did not meet the following GATK criteria: quality score > = 20, map quality score > = 40, FisherStrand score < = 40, HaplotypeScore <= 13, MQRankSumTest <= −12.5, ReadPosRankSum > −8. An additional “allele balance” filter based on the ratio of the number of reads supporting reference vs. alternate alleles in heterozygotes was implemented, where any loci with a balance ratio > 2.3 were filtered. The values of the above filters were decided based on the results of the success/failure rate of SNPs on two Affymetrix SNP arrays that were designed de novo to genotype ~50,000 SNPs in a test sample of 384 individuals. Quality metrics were plotted for successful vs. failed SNPs, and the quality cutoffs chosen visually based on scores that would have optimized the yield of successful SNPs while eliminating failed SNPs. We then filtered the SNP table to 519,902 genic SNPs (annotated as synonymous or non-synonymous [37, 41]) for which at least 10 % of individuals were genotyped and for which heterozygosity was no greater than 0.7. Following the filtering of SNPs, we removed six individuals in the bottom one percentile of the number of SNPs genotyped. Finally, we removed seven individuals who were outliers for inbreeding coefficient (range of 0.38 to 0.57), as determined using 221,950 synonymous SNPs from the original dataset and the R package ‘SNPRelate’ [42].

The common garden experimental design and methods for phenotyping total biomass are described in [43].

Species ancestry between white spruce and Engelmann spruce was estimated with unsupervised analysis in ADMIXTURE, using default parameters [44]. The present dataset contained sufficient sampling within the allopatric ranges of white spruce and Engelmann spruce to estimate ancestry from these species. We also estimated ancestry from Sitka spruce (*Picea sitchensis*), a parapatric congener, and removed 23 individuals whose value was estimated to be greater than 12% (the average percent expected from a double-backcross). To provide reference genotypes for Sitka spruce, similar sequence capture data from 26 pure Sitka spruce individuals and four white spruce x Sitka spruce hybrids were obtained from an unrelated study (Joane Elleouet, unpublished data). A subset of 289,987 SNPs was used to estimate ancestry, which were selected on the basis of low linkage with other SNPs, having a minor allele frequency > 0.01, and having <30% missing data across both the present dataset and the Sitka spruce data.

### Amino acid genotypes and PROVEAN

**Fig. 1:**
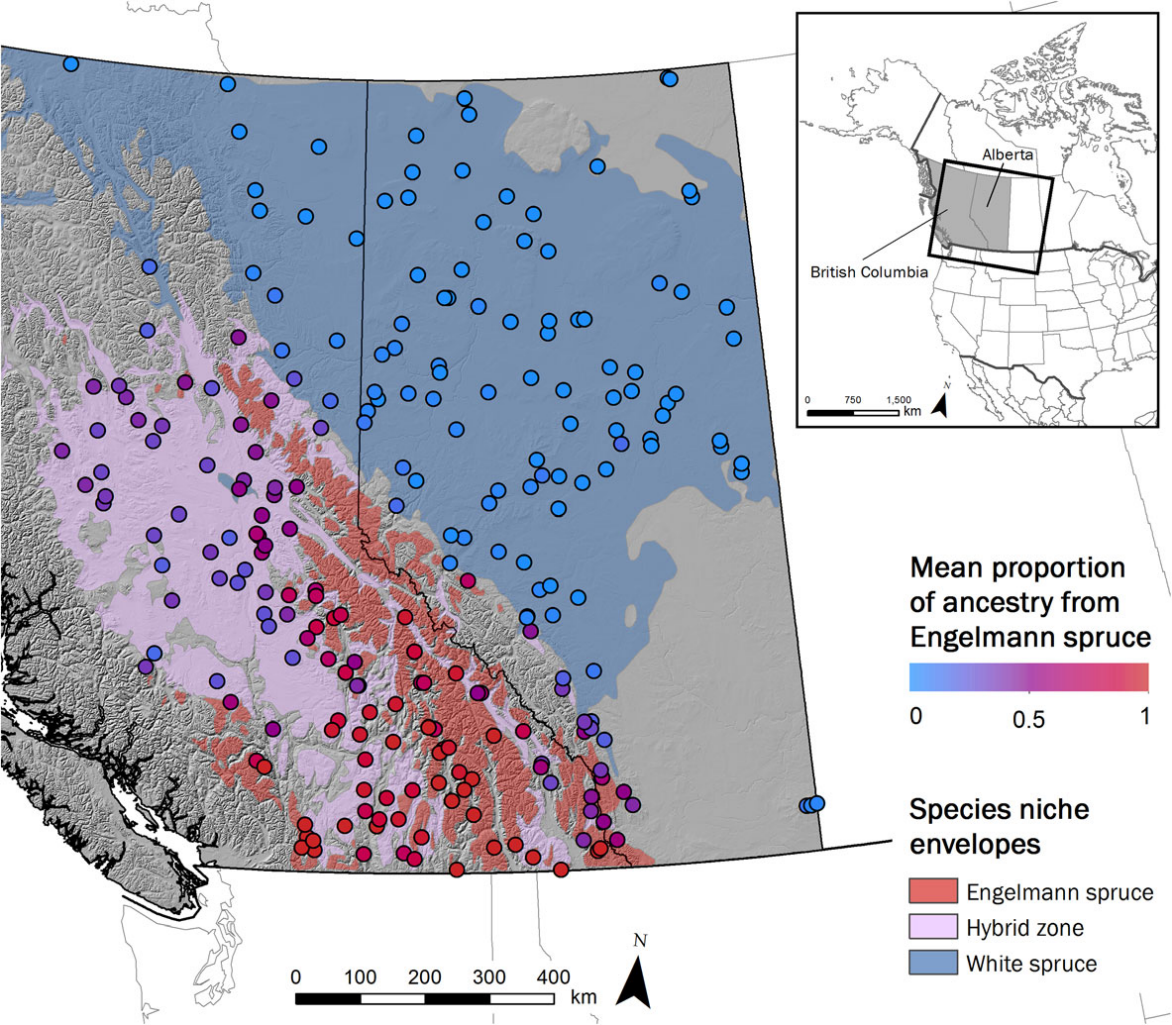
Sample collection locations. Seed was collected from the 249 locations indicated across British Columbia and Alberta, Canada. The average ancestry proportion of individuals in a given collection location is indicated by point color which ranges from blue, representing pure white spruce, to red, representing pure Engelmann spruce. Background colors show predicted species ranges (based on climatic niche model) of white spruce (blue), Engelmann spruce (red) and hybrids (purple). Niche envelopes were generated by Tongli Wang (unpubl.) with methodology as described in Wang et al. [57]. All maps were generated by JD, and produced using ESRI ArcGIS 10.2.2. No copyright permissions were required

In 539 trees from 247 locations (Fig. 1), we checked for amino acid variation resulting from 437,639 SNPs in 10,196 coding regions having complete open reading frame predictions [37, 41]. We constructed individual codon genotypes at each codon containing one or more SNPs using the reference transcriptome [41] for all monomorphic positions and SNP calls for polymorphic positions. We then translated codon genotypes at polymorphic codons to amino acid genotypes using the R package ‘Biostrings’ [45]. We removed variants with fewer than 500 alleles genotyped. Variants involving stop codons (i.e. variants at stop positions and nonsense variants) were not considered further in the present study. We calculated allele frequencies of amino acid variants. Polymorphic amino acid positions having more than one major allele (i.e. two or more alleles of equally high frequency) were removed from the dataset. We were left with 165,576 amino acid variants at 153,961 positions in 6928 proteins.

We translated all genes/predicted open reading frames containing amino acid variation to protein sequences using ‘Biostrings’. To avoid reference bias [21], we replaced the reference allele with the major allele at polymorphic amino acid positions (the reference allele was different from the major allele in about ~1% of polymorphic amino acid positions) and tested the minor allele for a deleterious signature. Testing only minor alleles for a deleterious signature was also justified for this study because our major alleles are ‘major’ across two species and are therefore, unlikely to be truly deleterious. While deleterious alleles may occasionally rise to high frequencies within populations at non-equilibrium conditions, it is very unlikely that the same allele would do so in each of two species. We confirmed that the vast majority (99.3%) of the relatively small number of major alleles that would have been flagged as deleterious (*n* = 1328, as indicated by PROVEAN score of minor allele > +2.282), were indeed major alleles in both species. Rather than deleterious, these alleles may actually be beneficial, but abnormal enough to receive a low PROVEAN score.

We used the program PROVEAN [16] to predict whether or not minor amino acid variants were deleterious. PROVEAN assumes that changes are more likely to be deleterious when they are more abnormal, with scores being lower when amino acid replacements are rarely seen among homologous protein sequences and their types rarely seen in conserved protein sequences. This is achieved using differences in the alignment score between the reference protein with and without the mutation of interest, to homologous proteins in the NCBI NR database using BLASTP. Alignment scores are derived from a BLOSUM substitution matrix [46], which gives the log-odds for every possible amino acid substitution based on the relative frequencies of amino acids and their observed substitution probabilities in very conserved regions of protein families in the BLOCKS database. Thus, we chose this program because the severity of amino acid mutations is empirically estimated using conserved protein sequences that are subject to purifying selection. Importantly, PROVEAN does not use information about allele frequency in its calculations. We used a threshold PROVEAN score below which variants were predicted to be deleterious of −2.282. This value was empirically determined to result in the highest balanced accuracy (78.75, based on a sensitivity of 78.39 and a specificity of 79.11) in tests of known disease variants and common polymorphisms (assumed to be neutral) in the “Human Polymorphisms and Disease Mutations” dataset by [16] (see the Discussion regarding the accuracy of specificity estimates). This balanced accuracy has been shown to be very similar to that of other programs available for predicting deleterious alleles (tested using similar methods), including SIFT, PolyPhen2 and Mutation Assessor [16]. Furthermore, studies that have used both PROVEAN and SIFT have found that the program used did not qualitatively affect the results [23, 25]. Finally, here we compare the relative strength of mutation load across populations, rather than estimating the absolute strength of mutation load. Thus, while we recognize that some fraction of the predicted deleterious alleles are likely false positive, the specific rate of false positives should not qualitatively affect our results, as we do not expect false positives to differ in average frequency across populations.

### Nucleotide diversity

To determine nucleotide diversity in the coding regions we implemented a modified version of the SNP calling pipeline in [37] (see Supplementary Methods). Briefly, we filtered SNPs using Qual scores and genotype quality scores over 20, and map quality scores of 40 and filtered all sites, including non-variable sites, with a minimum depth threshold of five for each individual. Using custom perl scripts we created corresponding fasta files for each individual and included “N” at sites that failed the filtering criteria for individuals. We extracted coding sequences for each coding region from these fasta alignments based on identified open reading frames for the transcripts [47].

To assess nucleotide polymorphism for each species, we used a modified version of Polymorphorama perl script [48–50] to generate summary statistics for each gene [47]. We estimated the number of synonymous sites, non-synonymous sites, and average pairwise diversity at synonymous (π_s_) and non-synonymous sites (π_n_). We only examined variable coding regions with more than 100 bp sequenced (with no missing data) in 20 or more individuals in each species (hybrid, white and Engelmann) leaving coding regions for 3531 separate genes. We used the non-parametric Kruskal-Wallis rank sum test to determine if the species differed for these sequence diversity statistics and the Nemenyi-tests (R package PMCMR) for multiple comparisons when significance was detected.

## Results

### Proportion of alleles predicted to be deleterious

We identified 291,892 polymorphic codon positions in 539 individuals of white spruce, Engelmann spruce and their hybrids (Fig. 1) in a previously reported exome capture dataset [36]. Of these, 126,316 coded for the same amino acid (which we refer to as synonymous) and 165,576 resulted in amino acid variants. The mean PROVEAN score of minor amino acid variants was −0.775 (0.004 SE) (Additional file 1: Figure S1). 13.35% of minor variants had PROVEAN scores below the threshold of −2.282, and were therefore predicted to be deleterious (Additional file 1: Figure S1). On average, individuals carried a heterozygous putative deleterious minor allele at 0.4% (0.001% SE) of polymorphic loci (note that from here forward, ‘polymorphic’ refers to the amino acid level) and were homozygous for putative deleterious minor alleles at 0.05% (0.0003% SE) of polymorphic loci. Also from here forward, we often refer to ‘predicted deleterious’ variants as simply ‘deleterious’ and ‘predicted non-deleterious’ variants as ‘non-deleterious’.

### The efficacy of PROVEAN predictions in spruce

**Fig. 2:**
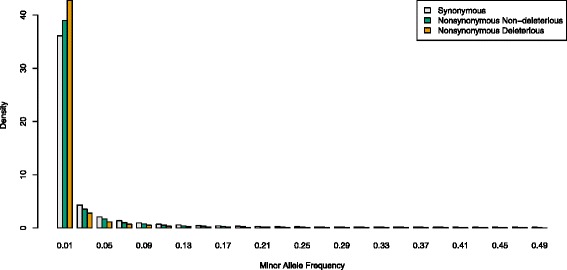
Folded site frequency spectrum. Synonymous minor alleles are shown in grey, nonsynonymous non-deleterious minor alleles are shown in green and nonsynonymous deleterious minor alleles are shown in orange

Variants predicted to be deleterious were at significantly lower allele frequencies on average than both synonymous codons (*p* < 10^−15^ based on Mann–Whitney *U* test) and variants predicted to be non-deleterious (p < 10^−15^ based on Mann–Whitney *U* test) (Fig. 2). To ensure that this result was not dependent on the PROVEAN score threshold chosen, we varied the threshold between −1 and −6 and found that the difference persisted across all values (data not shown). With lower thresholds, the magnitude of allele frequency differences increased but power to detect these differences decreased as sample size of predicted deleterious alleles decreased.

Because deleterious variants tend to be kept at low frequencies by purifying selection, we did not use a minor allele frequency cutoff in this study. However, some rare variants may be sequencing or genotyping errors rather than real variants, and these errors may appear deleterious more often than real variants since they are more likely to cause unusual amino acid changes and have never been exposed to natural selection. To address this issue, we removed all variants observed only once from the dataset (29,900 synonymous and 52,176 non-synonymous) and found that qualitative patterns of differences in allele frequency persisted (*p* < 10^−15^ based on Mann–Whitney *U* test for deleterious variants vs. synonymous codons, and *p* < 10^−15^ based on Mann–Whitney *U* test for deleterious vs. non-deleterious variants) (Additional file 1: Figure S2).

### Effect of mutation load on a fitness proxy

**Fig. 3:**
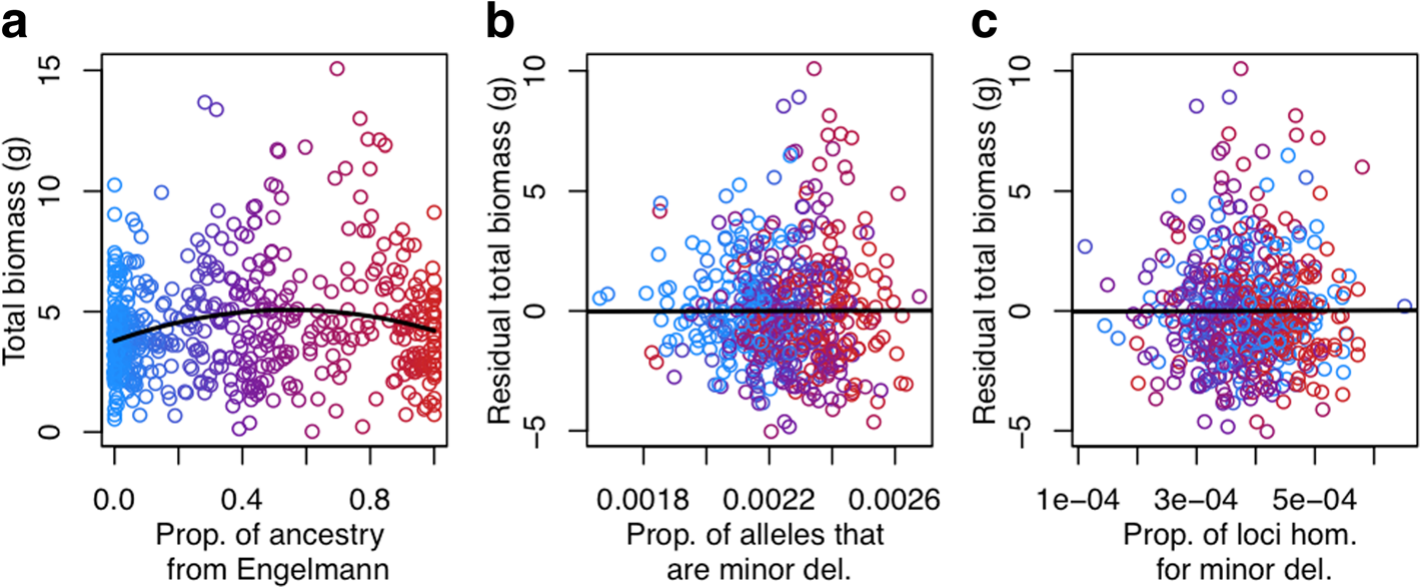
Effect of deleterious alleles on a fitness proxy**.** Quadratic regression of the proportion of ancestry from Engelmann spruce on total biomass (**a**) and linear regressions of those residuals on the proportion of alleles at polymorphic loci predicted to be deleterious (**b**) and the proportion of polymorphic loci predicted to be homozygous deleterious (**c**). The proportion of ancestry from Engelmann is represented by a color gradient with warm colors indicating a high proportion and cool colors indicating a low proportion

We found that the proportion of ancestry from Engelmann spruce, hereafter ‘ancestry proportion’, explained significant variation in total biomass of seedlings (dry root weight + shoot weight) of individuals, in a quadratic regression (*r* ^2^ = 0.04, F_2,533_ = 11.24, *p* = 1.7 × 10^−5^) (Fig. 3). After accounting for species identity, we found no effect of either the proportion of alleles at polymorphic loci that were predicted to be deleterious (F_1,534_ = 0.004, *p* = 0.95) or the proportion of polymorphic loci predicted to be homozygous deleterious (F_1,534_ = 0.005, p = 0.95) on total biomass (Fig. 3). However, we did find that the seven individuals who were likely recently inbred, as inferred from their unusually high inbreeding coefficients (range of 0.38 to 0.57), had significantly lower total biomass than other individuals (*t* = 3.1, df = 6, *p* = 0.02) (Additional file 1: Figure S3), providing support for use of total biomass as a fitness proxy.

### Deleterious load carried by individuals

**Fig. 4:**
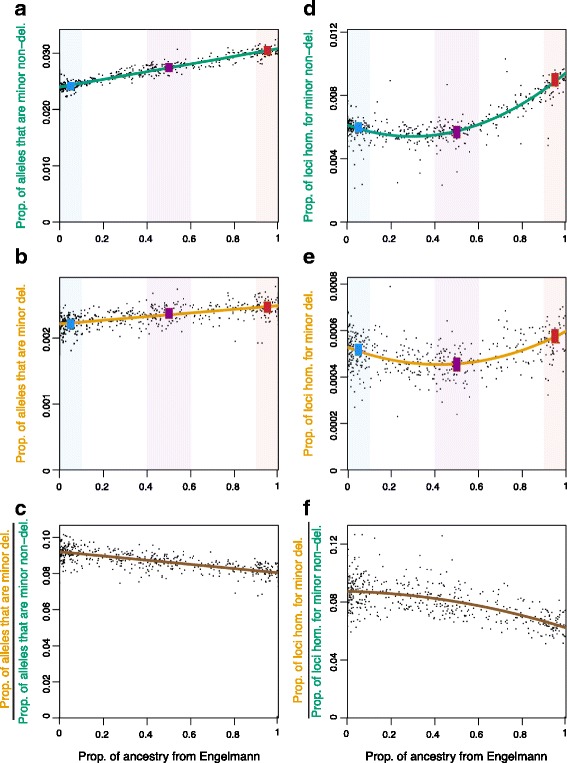
Prevalence of non-deleterious and deleterious minor alleles per individual by ancestry proportion. The proportion of ancestry from Engelmann spruce is shown against the proportion of alleles at polymorphic loci that are non-deleterious minor alleles (**a**) the proportion of alleles at polymorphic loci that are deleterious minor alleles (**b**), the ratio of the proportion of alleles at polymorphic loci that are deleterious minor alleles to the proportion of alleles at polymorphic loci that are non-deleterious minor alleles (**c**), the proportion of polymorphic loci that are homozygous for a non-deleterious minor allele (**d**), the proportion of polymorphic loci that are homozygous for a deleterious minor allele (**e**) and the ratio of the proportion of polymorphic loci that are homozygous for a deleterious minor allele to the proportion of polymorphic loci that are homozygous for a non-deleterious minor allele (**f**). Lines in in (**a**) - (**c**) represent linear regressions and those in (**d**) - (**f**) represent quadratic regressions. Vertical colored bars represent 95% confidence intervals for the mean of each species groups (blue for pure white spruce, red for pure Engelmann spruce and purple for intermediate hybrid) and columns of the corresponding background colors indicate the range of individuals included in each species group. Note difference in Y-axis scale among panels, especially for deleterious and non-deleterious alleles

Below, to be concise, we refer to the ‘proportion of alleles at polymorphic loci’, as simply the ‘proportion of alleles’ and the ‘proportion of polymorphic loci’ as simply the ‘proportion of loci’. There was a strong positive linear relationship between the proportion of ancestry from Engelmann spruce and both the proportion of alleles that are non-deleterious minor alleles (*r* ^2^ = 0.93, F_1,537_ = 6943, *p* < 10^−15^) and the proportion of alleles that are deleterious minor alleles (*r* ^2^ = 0.39, F_1,537_ = 337.3, p < 10^−15^) (Fig. 4a, b). We also tested for differences among binned species groups of ‘pure Engelmann spruce’ (i.e. proportion of ancestry from Engelmann spruce ≥90%), ‘pure White spruce’ (i.e. proportion of ancestry from Engelmann spruce ≤10%), and ‘intermediate hybrids’ (i.e. 40% ≤ proportion of ancestry from Engelmann spruce ≤60%) (Additional file 1: Figure S5). Both the proportion of minor alleles that are non-deleterious and the proportion of minor alleles that are deleterious were highly significantly different among these binned species groups (F_2,357_ = 2444.2, *p* < 10^−15^; F_2,357_ = 131.79, *p* < 10^−15^, respectively). On average, Engelmann spruce individuals carried 26% more non-deleterious minor alleles than white spruce individuals (Tukey HSD *p* = 0) and 12% more deleterious minor alleles than white spruce individuals (Tukey HSD p = 0) (Fig. 4a, b). Intermediate hybrids had intermediate values, with 14% more non-deleterious minor alleles than white spruce individuals (Tukey HSD p = 0) and 10% fewer non-deleterious minor alleles than Engelmann spruce individuals (Tukey HSD p = 0). Intermediate hybrids also had 7% more deleterious minor alleles than white spruce individuals (Tukey HSD p = 0) and 4% fewer deleterious minor alleles than Engelmann spruce individuals (Tukey HSD *p* = 5.7 × 10^−6^) (Fig. 4a, b). Qualitatively, the same patterns were found for mean allele frequencies of non-deleterious and deleterious minor alleles among the binned species group (Additional file 1: Figure S6). Furthermore, we tested how the ratio of the proportion of alleles that are minor and deleterious (response variable in Fig. 4b) to the proportion of alleles that are minor and non-deleterious (response variable in Fig. 4a) changed with the proportion of ancestry from Engelmann spruce. We found that this ratio decreased significantly with increasing ancestry from Engelmann spruce (*r* ^2^ = 0.53, F_1,537_ = 598.9, *p* < 10^−15^) (Fig. 4c).

Both the proportion of loci that were homozygous for a non-deleterious minor allele and the proportion that were homozygous for a deleterious minor allele increased with the proportion of ancestry from Engelmann spruce with positive quadratic curvature (*r* ^2^ = 0.77, F_2,536_ = 887.4, *p* < 10^−15^; *r* ^2^ = 0.29, F_2,536_ = 108.1, *p* < 10^−15^, respectively) (Fig. 4d, e). The proportion of loci that were homozygous for a non-deleterious minor allele and the proportion that were homozygous for a deleterious minor allele were both highly significantly different among binned species groups (F_2,357_ = 579.19, *p* < 10^−15^; F_2,357_ = 66.721, *p* < 10^−15^, respectively). On average, Engelmann spruce individuals had a 51% higher proportion of loci that were homozygous for a non-deleterious minor allele and a 11% higher proportion of loci that were homozygous for a deleterious minor than white spruce individuals (Tukey HSD *p* = 0 for both) (Fig. 4d, e). Intermediate hybrids had the lowest values, having a 5% lower proportion of loci that were homozygous for a non-deleterious minor allele than white spruce individuals (Tukey HSD *p* = 0.011) and 37% lower proportion of these than Engelmann spruce individuals (Tukey HSD *p* = 0). Intermediate hybrids had a 12% lower proportion of loci that were homozygous for a deleterious minor allele than white spruce individuals (Tukey HSD *p* = 0) and 21% lower proportion of these than Engelmann spruce individuals (Tukey HSD *p* = 0) (Fig. 4d, e). The ratio of the proportion of loci that were homozygous for a deleterious minor allele (response variable in Fig. 4e) to proportion that were homozygous for a non-deleterious minor allele (response variable in Fig. 4d) decreased significantly with increasing ancestry from Engelmann spruce with negative quadratic curvature (*r* ^2^ = 0.46, F_2,536_ = 228.3, *p* < 10^−15^) (Fig. 4f).

We had more white-like individuals than Engelmann-like individuals in our sample. This uneven sampling may have caused alleles at higher relative frequency in white than in Engelmann spruce to be deemed as major alleles, and therefore not tested for deleterious signatures. Thus, we estimated the number of major alleles that would have been minor alleles if we had even sampling. We did this by counting the number of major alleles at higher relative frequency in the white spruce binned group than in the Engelmann spruce binned group, and for which the relative frequencies of the two groups sum to less than one. We found only three of these alleles in the dataset and therefore, conclude that uneven sampling has had a minimal effect on our results.

### Nucleotide diversity

We found that average pairwise diversity at synonymous sites (π_s_, *χ* ^*2*^ = 71.54, df = 2, *p* < 0.001) and non-synonymous sites (π_n_)(*χ* ^*2*^ = 57.65*,* df = 2, *p* < 0.001) were significantly different among the binned species groups. White spruce had significantly lower π_s_ (mean = 0.0033, SE = 9.7 × 10^−05^) than both Engelmann (mean = 0.0040, SE = 1.0× 10^−04^) and intermediate hybrids (mean = 0.0040, SE = 1.0 × 10^−04^, *p* < 0.001), but Engelmann and intermediate hybrids did not differ in their average pairwise diversity at synonymous sites. This same pattern of significance was also repeated for non-synonymous sites (π_n_) (mean ± SE: white = 0.0012 ± 4.1 × 10^−05^, Engelmann = 0.0014 ± 4.4e-05, intermediate hybrid = 0.0014 ± 4.4 × 10^−05^).

## Discussion

We used the distribution of putatively deleterious alleles carried by individuals to infer the relative strength of mutation load across a natural hybrid complex. This approach allowed us to test the prediction that mutation load is reduced in hybrids that are locally adapted to intermediate environments, a pattern that confounds our ability to test this prediction phenotypically.

### Proportion of alleles predicted to be deleterious

Approximately 13% of nonsynonymous minor alleles segregating in the interior spruce hybrid complex were flagged as deleterious (using the threshold PROVEAN score recommended by [16]). This estimate is not far from other such estimates in wild populations, including about 20% in *Arabidopsis thaliana* and rice [17], about 12% in *Helianthus annuus* [23] and about 28% in *Populus trichocarpa* [25]. Here, we refrain from comparing numbers or proportions of deleterious alleles per individual (reflecting the extent of mutation load) with those estimated for other taxa, due to differences in methods and cautions explained below. However, inbreeding experiments have long suggested that conifers suffer from relatively high mutation load [32, 34, 35, 51, 52].

We recommend caution when interpreting absolute numbers and absolute proportions of deleterious alleles estimated bioinformatically in this and other studies. Choi et al. [16] suggest that PROVEAN has approximately an 80% specificity (meaning that 20% of all true negative SNPs tested would give false positive results). Surprisingly, this and other studies have found a lower proportion of variants that are flagged as deleterious (a group that should include true positives + false positives) than are expected or even possible given the estimated specificity of PROVEAN (or the other similar programs that have been used) [17, 20, 22, 23, 25]. Finding fewer positive results than predicted by the expected false positive rate is difficult to explain unless the stated specificity of the PROVEAN method is estimated in error.

To accurately estimate specificity, one must test the focal program on a set of known neutral alleles and calculate the number of false positives generated. However, confirming neutrality is very difficult. Studies estimating specificity (including Choi et al. [16]) tend to deem disease-causing variants as truly deleterious and variants not known to cause disease as truly neutral. We predict that many of the variants assumed to be truly neutral are actually weakly deleterious, with effects too small to detect phenotypically or through functional assays, but large enough to be kept at low frequency by selection. If true, this would help to explain why studies like ours often find fewer false positives than predicted by specificity estimates. While this issue calls into question direct interpretation of bioinformatically estimated numbers and proportions of deleterious alleles, such estimates are still useful for relative comparisons across groups within studies (e.g., across the interior spruce hybrid complex) and across studies that use the same PROVEAN score threshold or for which the programs used have similar sensitivity and specificity, estimated in a similar way.

### The efficacy of PROVEAN predictions in spruce

PROVEAN and similar programs predict that alleles are deleterious when they occur in conserved amino acid positions and when the amino acid replacement is either a relatively rare or causes a substantial biochemical change at the site [12–16]. However, in some cases such alleles may not be truly deleterious. These alleles may represent genetic innovations that are globally beneficial to the focal species, or they may be beneficial in particular environments and neutral or deleterious in others. In support of the PROVEAN predictions, however, we find strong evidence that alleles predicted to be deleterious are at lower allele frequencies on average than those not predicted to be deleterious, suggesting that predicted deleterious alleles are enriched for truly deleterious alleles. We also find that non-synonymous mutations that were not predicted to be deleterious were at lower allele frequencies than synonymous mutations, most likely reflecting the presence of false negatives in that category. Nonetheless, similar evidence that predicted deleterious alleles are enriched for truly deleterious alleles has recently been found in other systems as well, including *Arabidopsis* [17], maize [20], barley and soybean [22], sunflower [23] and humans [19, 21, 24], suggesting that that these approaches are useful for identifying deleterious alleles underlying mutation load.

Genotyping error presents an underappreciated complication to studies identifying deleterious alleles bioinformatically. Because we expect deleterious alleles to be at low frequencies, we cannot use a minor allele frequency cutoff when filtering SNPs to help eliminate rare genotyping errors, as is typically done in studies of the genetics of adaptation. Because genotyping errors are not real alleles that are exposed to selection, they may appear to be alleles resulting in more severe biochemical changes and thus be called deleterious more often than real alleles. This represents an alternative explanation for the commonly reported pattern that predicted deleterious alleles are at lower frequencies. However, when we eliminated apparent alleles observed only a single time (the frequency class most likely to contain genotyping errors), we still found strong evidence that predicted deleterious alleles are at lower allele frequencies. This suggests that while rare genotyping errors are almost certainly present in the dataset, they are not driving the pattern, and instead, many real deleterious alleles have been identified.

Ideally, we would like to confirm that predicted deleterious alleles indeed have a negative effect on a phenotypic fitness proxy. Because most deleterious alleles are of small effect and are at low allele frequencies, detecting their individual phenotypic effects requires prohibitively massive sample sizes. Here, we tested for cumulative effects of deleterious alleles (i.e. the proportion of alleles that are deleterious, or the proportion of loci that are homozygous deleterious) on the total biomass of seedling individuals, a proxy for juvenile fitness, and we were not able to detect an effect of either variable beyond the effects of ancestry proportion itself. Because deleterious alleles tend to be strongly differentiated among species, their patterns are tightly correlated with those of ancestry. This has perhaps led to low power for detecting an additional effect of mutation load on our phenotypic fitness proxy. Moreover, seedling biomass is only one small component of total fitness and the correlation between this trait and fitness may not be strong. Finally, our sample of putatively deleterious alleles is likely only a small fraction of those that exist in the genome, and many may be outside of coding regions, which our approach could not target. Zhang et al. carried out a similar test in *Populus trichocarpa,* and they did detect a significant effect of the proportion of putatively deleterious homozygous alleles on plant height after accounting for distance from the range center and population structure using principal components analysis [25]. Other studies have also found that genes associated with complex traits or genes with known functional effects are enriched for bioinformatically predicted deleterious variants [17, 20, 25].

### Relative amount of mutation load in white spruce and Engelmann spruce

Our results suggest that Engelmann spruce carries a greater mutation load than white spruce. Engelmann spruce individuals tend to be burdened by more deleterious alleles (in both heterozygous and homozygous state) due to both higher diversity and higher frequencies of all rare alleles, including deleterious ones. Mitochondrial DNA haplotype diversity is also considerably greater in Engelmann spruce than in white spruce where their ranges overlap (JC Degner, unpublished data). Furthermore, isozyme diversity increases with latitude in Engelmann spruce, and it is highest where its range overlaps with white spruce, a pattern attributed to hybridization [29]. On the other hand, white spruce in this area are at the leading edge of a rapid and long distance range expansion [26, 27]. Therefore, their relatively low diversity may be due to serial founder events that have taken place during range expansion. It may seem counterintuitive that the species with greater genetic diversity has greater mutation load. This pattern could be explained by anything that maintains a larger species-level population size or a higher mutation rate. In particular, if local population sizes are low and there is low dispersal between local populations, then drift is strong relative to selection within local populations, allowing neutral and deleterious alleles to drift locally to high frequencies, while low dispersal maintains a high level of genetic diversity at the species level [53]. Engelmann spruce is adapted to high elevations and their range is currently fragmented on mountain tops [28]. It is plausible (although by no means certain) that during glaciation, refugial population sizes were small with low dispersal between them, contributing to the pattern we observe.

While with increasing ancestry from Engelmann spruce, individuals have more deleterious alleles on average, we also find that the same individuals have proportionately fewer deleterious alleles relative to non-deleterious alleles (both in total and in homozygous state). In other words, patterns of deleterious alleles vary with ancestry proportion when correcting for patterns in non-deleterious alleles. First, this provides evidence that patterns in deleterious alleles are distinguishable from those of demography (as represented by non-deleterious alleles). Second, it may provide evidence that selection is less efficient at removing deleterious variants in more white spruce-like populations, due to weaker purifying selection and/or stronger genetic drift. Given that these white spruce populations are at the leading edge of a recent, long-distance range expansion [26, 27], these results may be a signature of serial founder effects during range expansion (i.e. expansion load) [54]. However, further work is needed to test for expansion load in white spruce. Similarly, [55] found that while African American individuals contained more non-deleterious and deleterious variants than European American individuals, on average, they had a lower proportion of deleterious variants, likely resulting from both increased drift due serial founder effects and a decreased strength of purifying selection in populations that expanded out of Africa [24]. Note though, that several other relevant human studies have been done, with a range of results (e.g. [21, 24, 55, 56]. Together they are forming a detailed picture of the effects of the out-of-Africa expansion on deleterious variation in humans. Also importantly, while there are some similarities between our results and human results, there are also differences. These are likely due in part to significant differences in demographic history between the systems.

### Relative amount of mutation load in parental species and their hybrids

Hybrids are intermediate relative to parental species for the proportion of alleles that are deleterious. Thus, if most deleterious alleles have additive effects, then hybrids should have an intermediate mutation load relative to parental species. However, evidence suggests that deleterious alleles tend to be (at least partially) recessive [2, 7–9]. Here, we find that hybrids have a lower proportion of loci that are homozygous for deleterious alleles than either parental species. While this pattern is also expected (and was found) for non-deleterious alleles, showing a decrease in the homozygosity of deleterious alleles provides direct evidence for the possibility of complementation in hybrids. Complementation is the mechanism underlying the dominance hypothesis of heterosis [3, 4]. Even if complementation of deleterious alleles does not have a large enough effect to result in heterosis (i.e., hybrid fitness > parent fitness), it may still contribute to higher fitness in hybrids than they would otherwise have and therefore, have a significant impact on the outcomes of hybridization and on the stability of hybrid zones. When hybrids also benefit from local adaptation to their own environment, decreased mutation load due to complementation of deleterious alleles may give them a fitness advantage as well, though its effects on phenotypic fitness proxies would be inseparable from those of local adaptation. Studying mutation load at the genetic level has allowed us to infer that interior spruce hybrids, which are locally adapted to environmental conditions that are intermediate to the parental species (Engelmann and white spruce), also benefit from reduced mutation load due to complementation of deleterious alleles, given that most deleterious alleles are recessive.

## Conclusions

Here we showed that PROVEAN is a useful tool for identifying the genetic basis of mutation load in the interior spruce hybrid complex. The set of putatively deleterious alleles we identified allowed us to compare the relative strength of mutation load across the hybrid complex. We found that Engelmann spruce suffers from greater mutation load than white spruce due to higher frequencies of rare deleterious alleles. Given that deleterious alleles tend to be recessive, we also find that hybrids have lower mutation load than either of the parental species, due to complementation of deleterious alleles introduced by each of the parental species. Along with bounded hybrid superiority, this reduced mutation load likely contributes to the high hybrid fitness previously reported in this complex [31].

Interior spruce is an economically important species complex Canada, and is the second most planted tree type in British Columbia [31]. Because these trees suffer from high mutation load, understanding the genetic basis mutation load and factors that contribute to its strength will help us with management and breeding of this important genetic resource.

## Additional file

Additional file 1: Supplementary Material. Supplementary Figure S1-S6 (referenced in text). (PDF 9957 kb)

## Abbreviations

PCR: Polymerase chain reaction
SNP: Single nucleotide polymorphism
