# Supplementary material for: Bioinformatically predicted deleterious mutations reveal complementation in the interior spruce hybrid complex
Source: BMC Genomics. 2017 Dec 15;18:970. doi: 10.1186/s12864-017-4344-8 (PMC5731209; doi:10.1186/s12864-017-4344-8)
Supplement: Supplementary file 1 — Supplementary Material. Supplementary Figure S1-S6 (referenced in text). (PDF 9957 kb) [file 12864_2017_4344_MOESM1_ESM.pdf]

Supplementary Figures

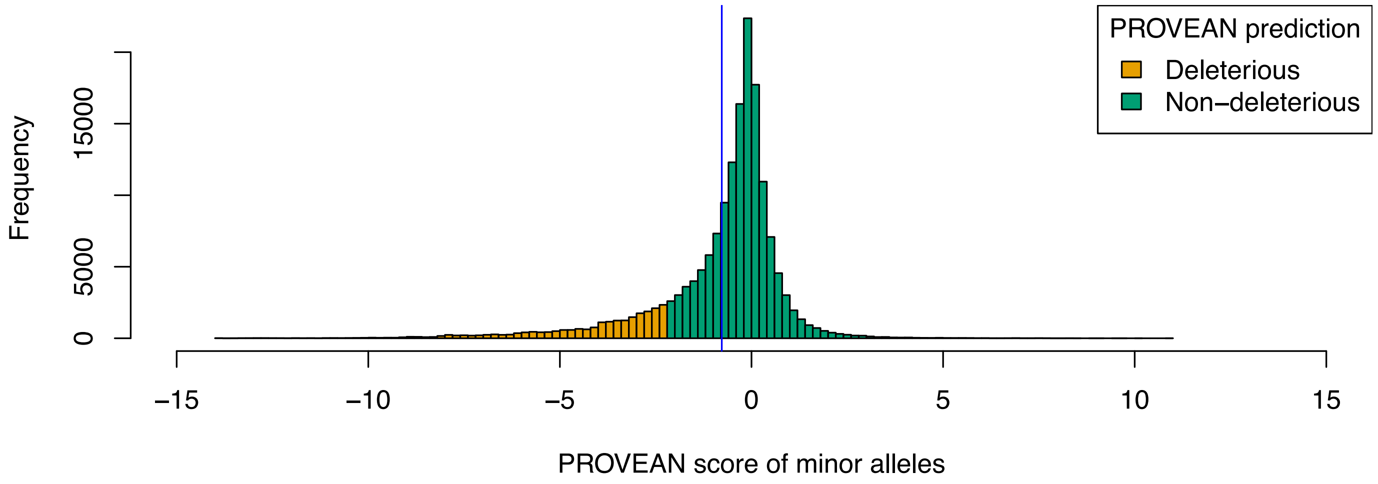

Figure S1 Distribution of PROVEAN scores for minor amino acid variants sampled from the population. The blue line is the mean score. Green bars represent variants predicted to be non-deleterious and orange bars represent variants predicted to be deleterious. All maps were generated by JD, and produced using ESRI ArcGIS 10.2.2. No copyright permissions were required.

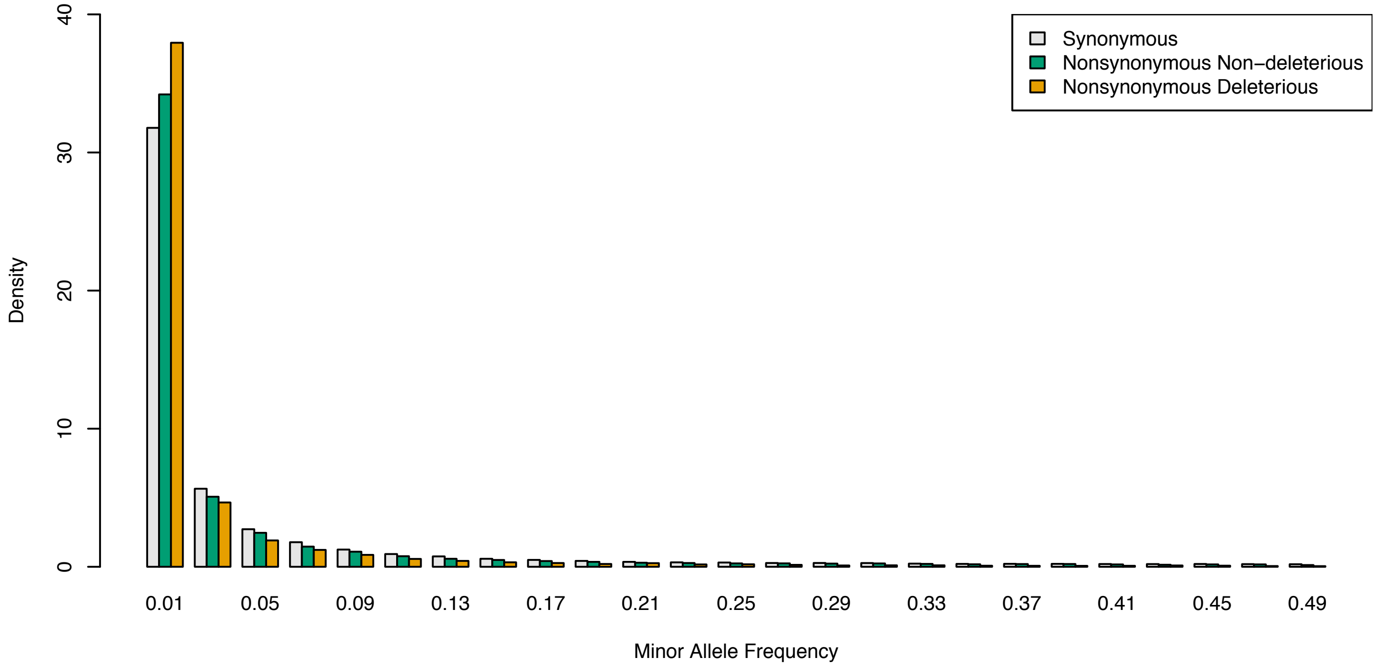

Figure S2 Folded site frequency spectrum, excluding variants observed only once. Synonymous minor alleles are shown in grey, nonsynonymous non-deleterious minor alleles are shown in green and nonsynonymous deleterious minor alleles are shown in orange.

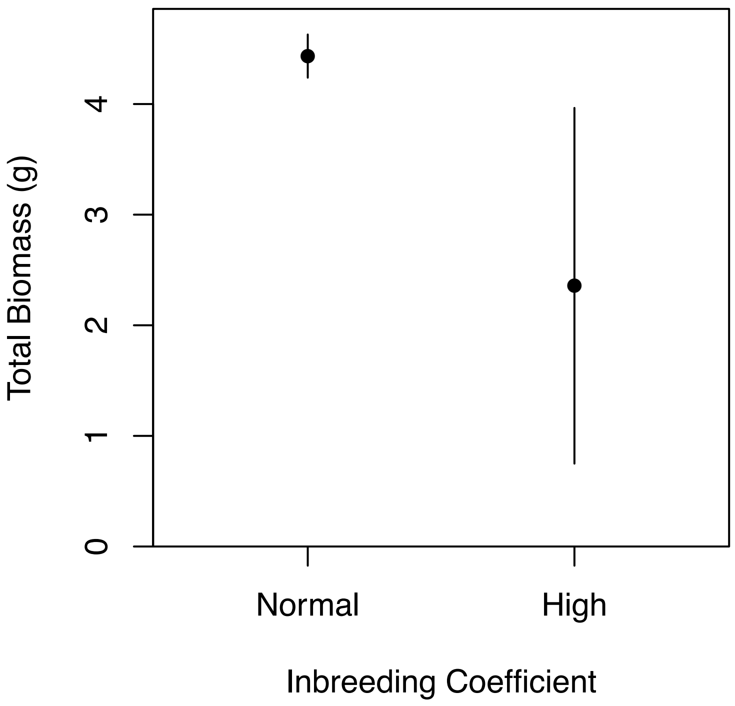

Figure S3 – Inbred individuals have lower total biomass (g). The mean total biomass of seven individuals who were outliers having high inbreeding coefficients, and were therefore likely recently inbred, is shown with the mean total biomass of the other 543 individuals in the distribution. Error bars are 95% confidence intervals of the mean.

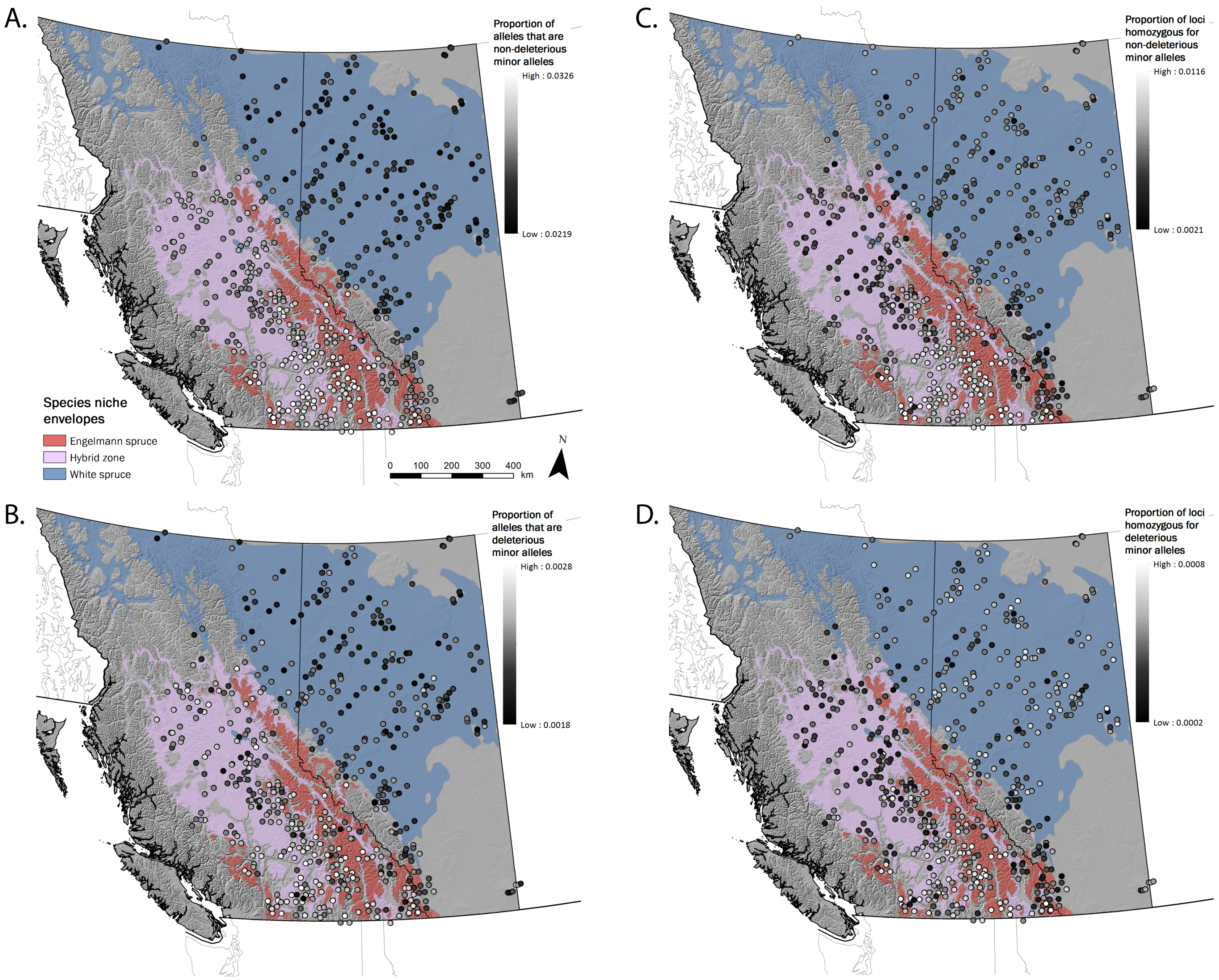

Figure S4 – Geography of the prevalence of non-deleterious and deleterious minor alleles per individual. Variables denoted by color gradients correspond with response variables in Figure 4 (A)(B)(D) and (E). Background colors show predicted species ranges (based on climatic niche model) of white spruce (blue), Engelmann spruce (red) and hybrids (purple). As indicated by the color gradient bar, cool colors represent low proportions and warm colors represent high proportions. Niche envelopes were generated by Tongli Wang (unpubl.) with methodology as described in Wang *et al.* (2016). Forest Ecol. Manag. 360: 357-366. All maps were generated by JD, and produced using ESRI ArcGIS 10.2.2. No copyright permissions were required.

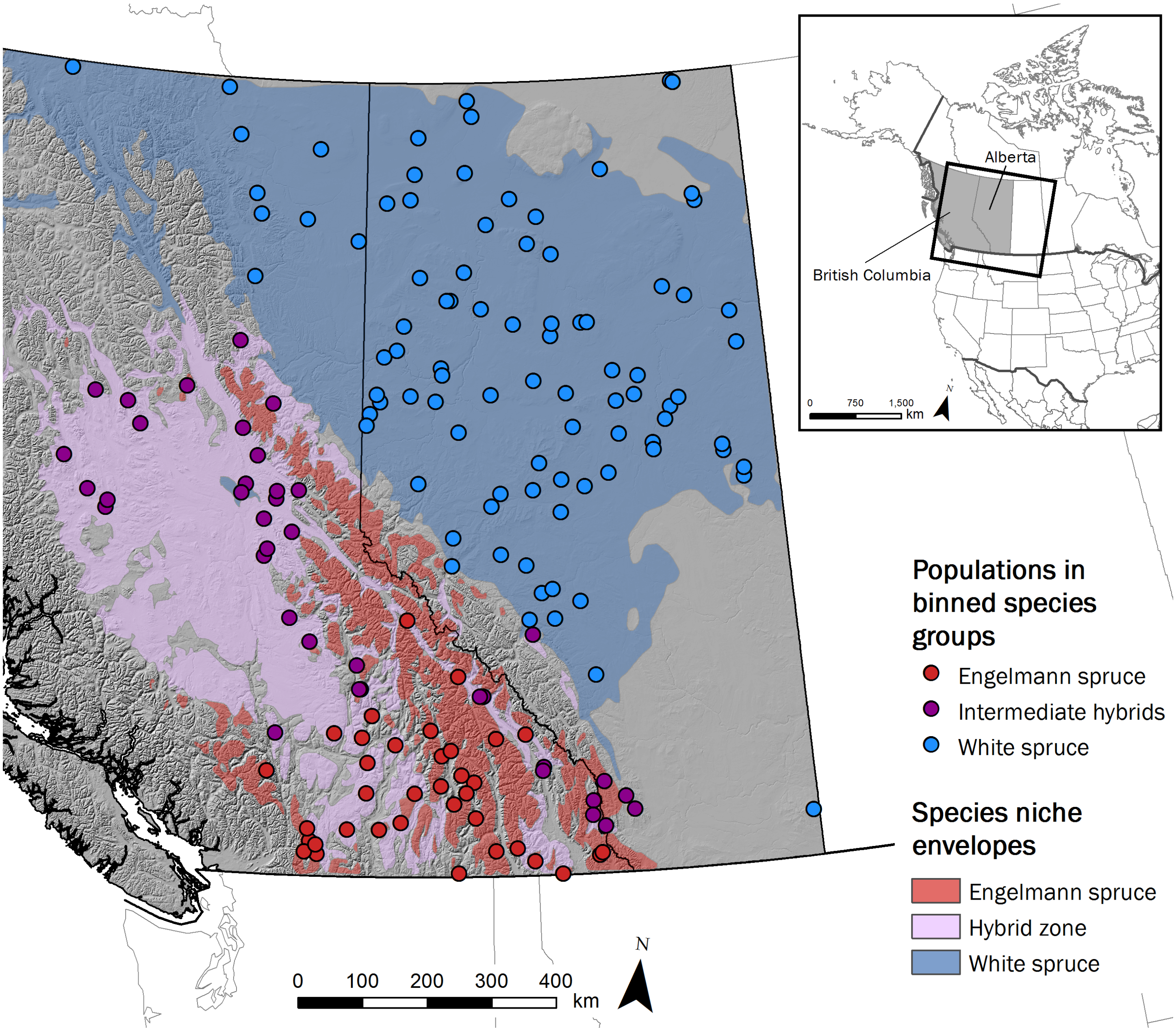

Figure S5 – Collection locations of individuals within binned species groups.
Pure white spruce individuals (i.e. proportion of ancestry from Engelmann ≤ 0.1) are represented by blue dots, pure Engelmann spruce individuals (i.e. proportion of ancestry from Engelmann ≥ 0.9) are represented by red dots and intermediate hybrid individuals (i.e. 0.4 ≤ proportion of ancestry from Engelmann ≤ 0.6) are represented by purple dots. Background colors show predicted species ranges (based on climatic niche model) of white spruce (blue), Engelmann spruce (red) and hybrids (purple). Niche envelopes were generated by Tongli Wang (unpubl.) with methodology as described in Wang *et al.* (2016). Forest Ecol. Manag. 360: 357-366. All maps were generated by JD, and produced using ESRI ArcGIS 10.2.2. No copyright permissions were required.

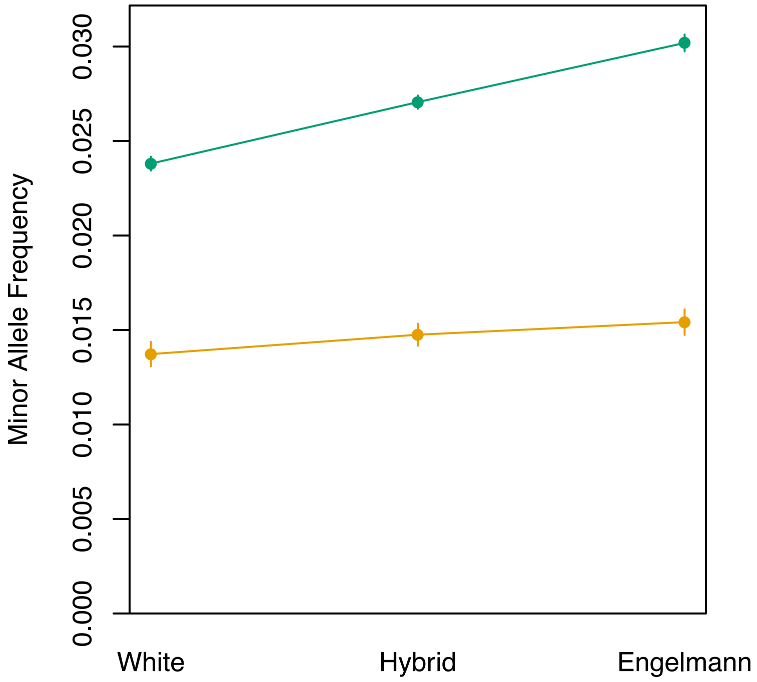

Figure S6 – Mean allele frequencies of non-deleterious and deleterious minor alleles among binned species groups. Non-deleterious minor alleles are shown in green and deleterious minor alleles are shown in orange. Error bars are 95% confidence intervals.
